# Epidemiological and genetic characteristics of EV71 in hand, foot, and mouth disease in Guangxi, southern China, from 2010 to 2015

**DOI:** 10.1371/journal.pone.0188640

**Published:** 2017-12-07

**Authors:** Minmei Chen, Yu Ju, Min Chen, Zhenguo Xie, Kaijiao Zhou, Yi Tan, Jianjun Mo

**Affiliations:** Institute of Acute Infectious Diseases Control and Prevention, Guangxi Zhuang Autonomous Region Center for Disease Prevention and Control, Nanning, Guangxi, China; Fudan University, CHINA

## Abstract

Hand, foot, and mouth disease (HFMD) is a significant public health challenge in China. *Human enterovirus 71* (EV71) is regarded as the predominant causative pathogen of HFMD. Since 2015, two inactivated EV71 vaccines have been approved in mainland China, and because their use could change the HFMD pathogen spectrum, this should now be monitored. However, the epidemiological and genetic trends of EV71 with respect to HFMD in Guangxi, southern China, are still not clear. In this study, we describe the epidemiological and genetic characterization of this virus in clinically-diagnosed HFMD reported from 2010 to 2015 in Guangxi. Data showed that a two-year epidemic cycle, with a predominance of EV71 infections, contributed to HFMD outbreaks in Guangxi. Furthermore, this virus is a major causative agent of severe and fatal HFMD. Interestingly, in Guangxi, EV71-positive rates tended to decrease over time. In particular, EV71-positive rates were found in Fangchenggang city, which reported very few severe and fatal cases over the six-year period. Phylogenetic analysis of the VP1 gene revealed that the major circulating strains belonged exclusively to genotype C, subtype 4a (C4a), and most clustered with strains circulating in southern China. The most interesting finding was that a strain isolated in 2012 clustered with Vietnamese strains isolated from 2011–2012. The data highlight the importance of pathogen surveillance for HFMD in China, especially Guangxi, which is located on the border of China and the Association of Southeast Asian Nations.

## Introduction

Hand, foot, and mouth disease (HFMD) is a common childhood infectious disease that is normally self-limiting; however, in few cases, there can be serious complications with the nervous and respiratory systems, which can lead to death. HFMD is a considerable global public health challenge, especially in the Asia-Pacific region [1]. Since 2008, the numbers of HFMD cases that have been reported by national disease control and prevention agencies have increased significantly in China, with a total of 344,688 cases, and 25 deaths, recorded from January 1 to May 10 of 2017.

HFMD is caused by members of the enterovirus family, among which *human enterovirus 71* (EV71) and *coxsackievirus A16* are the main pathogens responsible for global outbreaks [2, 3]. EV71 infection has been reported to be responsible for both severe and fatal HFMD [1, 4, 5]. On December 3, 2015, the Chinese Food and Drug Administration (CFDA) approved two inactivated EV71 whole-virus vaccines for preventing severe HFMD [6, 7]. Consequently, EV71 vaccination began in Guangxi from 2016 and continues to date. Because this might change the HFMD pathogen spectrum, monitoring EV71 epidemics at different times and geographical locations remains a high priority.

The Guangxi autonomous region is located in the south of China, and is divided into fourteen cities; it is one of the Chinese provinces that is most affected by HFMD [8]. In Guangxi, the number of reported cases has increased from 2008 to 2015, and since 2008, the incidence and rate of severe and fatal cases has consistently been the highest in China. In addition, from January 1 to May 10 of 2017, the number of reported cases (46,674) and incidence (97/10,000) were highest in Guangxi, among 31 Chinese provinces. Elucidating the epidemiological and evolutionary characteristics of E71 is critical for guiding the development of HFMD prevention and control strategies. This study focused on characterizing the epidemiological profiles and molecular evolution of EV71 in HFMD in the Guangxi autonomous region from January 2010 to December 2015. The results therefore provide information describing the baseline HFMD pathogen profile before vaccination was implemented.

## Materials and methods

### Ethics statement

This research was approved by the Human Research Ethics Committee of Disease Control and Prevention of Guangxi Zhuang Autonomous Region. Specimens were obtained from sentinel hospitals associated with the Center of Guangxi Zhuang Autonomous for Disease Control and Prevention (Guangxi CDC) for EV surveillance. It was determined by the National Health and Family Planning Commission of China that data collection for HFMD cases was part of continuous public health surveillance for a notifiable infectious disease and was thus exempt from institutional review board assessment. All patient information was kept anonymous to protect patient confidentiality.

### Case definitions

HFMD case-reporting criteria were based on the national guidelines for the control and prevention of HFMD (issued by the Ministry of Health in China (2009)). The diagnosis criteria for HFMD, severe cases, and laboratory test procedures followed the HFMD Clinical Diagnosis and Treatment Guidelines (2010). Mild cases were defined as the absence or presence of fever accompanied by a rash (maculopapule or vesicular rash) appearing on either the hand, foot, mouth, or buttock. Severe cases were defined the presence of at least one of the following complications: acute flaccid paralysis, aseptic meningitis, pulmonary edema, encephalitis, hemorrhage, or cardiopulmonary collapse. Clinically diagnosed cases, along with laboratory evidence of enteroviruses infection (including EV71, CA16, or other enteroviruses), were detected using real-time RT-PCR, and these were considered ‘laboratory-confirmed’ cases.

### Sample collection

There are 14 cities in Guangxi, and every city has set up sentinel sites responsible for the national surveillance program for HFMD. The diagnostic criteria for HFMD were defined by the Ministry of Health. Clinical specimens (including stool or rectal swabs and throat swabs) from patients were collected by sentinel practitioners and were transported to a pathogen laboratory. Meanwhile, a standardized report form was sent to the local CDC. A total of 44,284 clinical specimens were collected in the study from January 1, 2010 to December 31, 2015.

### Epidemiological analysis

Case information was aggregated at the municipal level. All statistical analyses were performed using R (R Foundation for Statistical Computing, Vienna, Austria; https://www.r-project.org/). Differences in age and pathogen distribution were analyzed using a chi-square test. The seasonal distribution of HFMD cases and EV71-positive cases was plotted as a line graph to detect peaks across the entire year. The spatial distribution of EV71-positive HFMD cases for each municipality was shown on maps created using maptools and ggplot2 in R. EV71 clusters were created using pheatmap in R. All statistical tests were two sided, and p values < 0.05 were considered statistically significant.

### Viral RNA extraction and detection for enteroviruses

Specimen processing was performed as previously described with a slight modification [9]. Stool suspensions were created by mixing 0.1 g of stool sample, 0.1 mL of chloroform, and 1 mL of phosphate-buffered saline. The suspensions were shaken vigorously for 20 min, followed by centrifugation at 3,000 × *g* for 5 min at 4°C. For rectal swab samples, the supernatant was prepared by centrifuging the fluid at 13,000 × *g* for 1 min and transferring the supernatant to a fresh tube. Viral RNA was extracted from 200 μL of supernatant using the viral RNA mini kit (Qiagen, Hilden, Germany) according to the manufacturer’s instructions. RNA from each sample was examined using real-time RT-PCR kits (Diagnostic Kit for human enteroviruses, EV71, and CA16, Jiangshu Shuoshi Biological Technology Co., Ltd, Taizhou, China) according to the manufacturer’s instructions. The protocol is provided as DOI: dx.doi.org/10.17504/protocols.io.jhzcj76.

### Virus isolation and sequencing

Viruses were isolated from cultured rhabdomyosarcoma (RD) cells and human epidermoid cancer cells (Hep-2) cells. After the cells were inoculated with virus-contaminated samples, they were cultured at 37°C and observed for three passages to ensure sufficient time for viral replication. When the cytopathic effect occurred for 75–100% of the cell monolayer, the virus was harvested. Collected viral particles were analyzed by RT-PCR and the complete VP1 gene sequences from EV71 and CA16 isolates were amplified as previously described [10]. The amplified products were sent to Sangon Biotech Co., Ltd (Shanghai, China) for DNA Sanger sequencing. The protocol is provided as DOI: dx.doi.org/10.17504/protocols.io.jh2cj8e. The VP1 sequences of the 57 EV71 strains have been submitted to GenBank (GenBank accession numbers MF185255–185311).

### Phylogenetic analyses

Evolutionary history was inferred using the neighbor-joining method [11]. The optimal tree with the sum of branch length = 1.07373051 was shown. The percentages of replicate trees in which the associated taxa clustered together in the bootstrap test (1000 replicates) were shown next to the branches. The tree was drawn to scale, with branch lengths given as the same units as those for the evolutionary distances used to infer the phylogenetic tree. Evolutionary distances were computed using the p-distance method, and the units were the number of base differences per site. The analysis involved 130 nucleotide sequences. The codon positions included were first, second, third, and non-coding positions. All positions containing gaps and missing data were eliminated. There were 881 positions in the final dataset. Evolutionary analyses were conducted using MEGA7 [12]. The phylogenetic tree was displayed using the online tool, Interactive Tree of Life (iTOL) [13]. The complete nucleotide sequences of 73 EV71 VP1 genes from GenBank were analyzed with the sequences of the VP1 genes obtained in this study (S1 Table).

## Results

### Demographic characteristics of HFMD epidemiology in Guangxi

A total of 44,284 clinically diagnosed HFMD cases were reported by the laboratories in Guangxi. Of these, 27,251 patients were male and 16,384 were female, with a male-to-female ratio of 1.67 (range: 1.59 to 1.72). Among these total HFMD cases, there were 8,797 severe cases and 363 fatal cases reported from 1 January 2010 to 31 December 2015. In Guangxi, HFMD epidemics were observed to occur in 2-year cycles; for comparison, the infection cycle has been reported to occur in 2–3 year cycles in Malaysia [14]. More severe and fatal cases occurred in years with large epidemics (including 2010, 2012, and 2014) compared to incidences in years with small epidemics (2011, 2013, and 2015).

Differences in HFMD incidence among the five different age groups were statistically significant (χ^2^ = 172.60, p < 0.001; Table 1). For this, the largest group was the 1–3-year old group, accounting for 52.40% of the cases; the second largest group was the ≤ 1-year old group, accounting for 32.41% of the cases. Most cases (96.62%) occurred in patients less than 5 years of age, which is consistent with the results of previous studies [15]. The distribution of HFMD cases was similar over the period of 2010–2015.

**Table 1:** Epidemiological characteristics of clinically reported and diagnosed hand, foot, and mouth disease (HFMD) cases in Guangxi, 2010–2015.

Variable	2010	2011	2012	2013	2014	2015
**HFMD Cases**						
Mild cases	2215	5296	6384	6378	7335	7335
Severe cases	1927	383	2063	417	3130	877
Fatal cases	107	21	103	11	111	10
Unknown	4	0	2	1	174	0
Total	4253	5700	8552	6807	10750	8222
**Gender**						
Male	2515	3576	5043	4254	6693	5049
Female	1536	2113	2936	2521	4048	3166
Unknown	202	11	573	32	9	7
Gender ratio	1.64	1.69	1.72	1.69	1.65	1.59
**Age Group (%)**						
≤ 1	34.40	29.96	30.30	34.93	30.81	35.29
1–3	48.50	53.80	52.80	51.92	54.24	50.99
3–5	12.90	12.82	12.80	9.99	12.08	10.70
5–10	3.41	2.96	3.54	2.76	2.62	2.78
> 10	0.79	0.46	0.56	0.40	0.24	0.24

### Temporal characteristics of HFMD epidemiology in Guangxi

**Fig 1:**
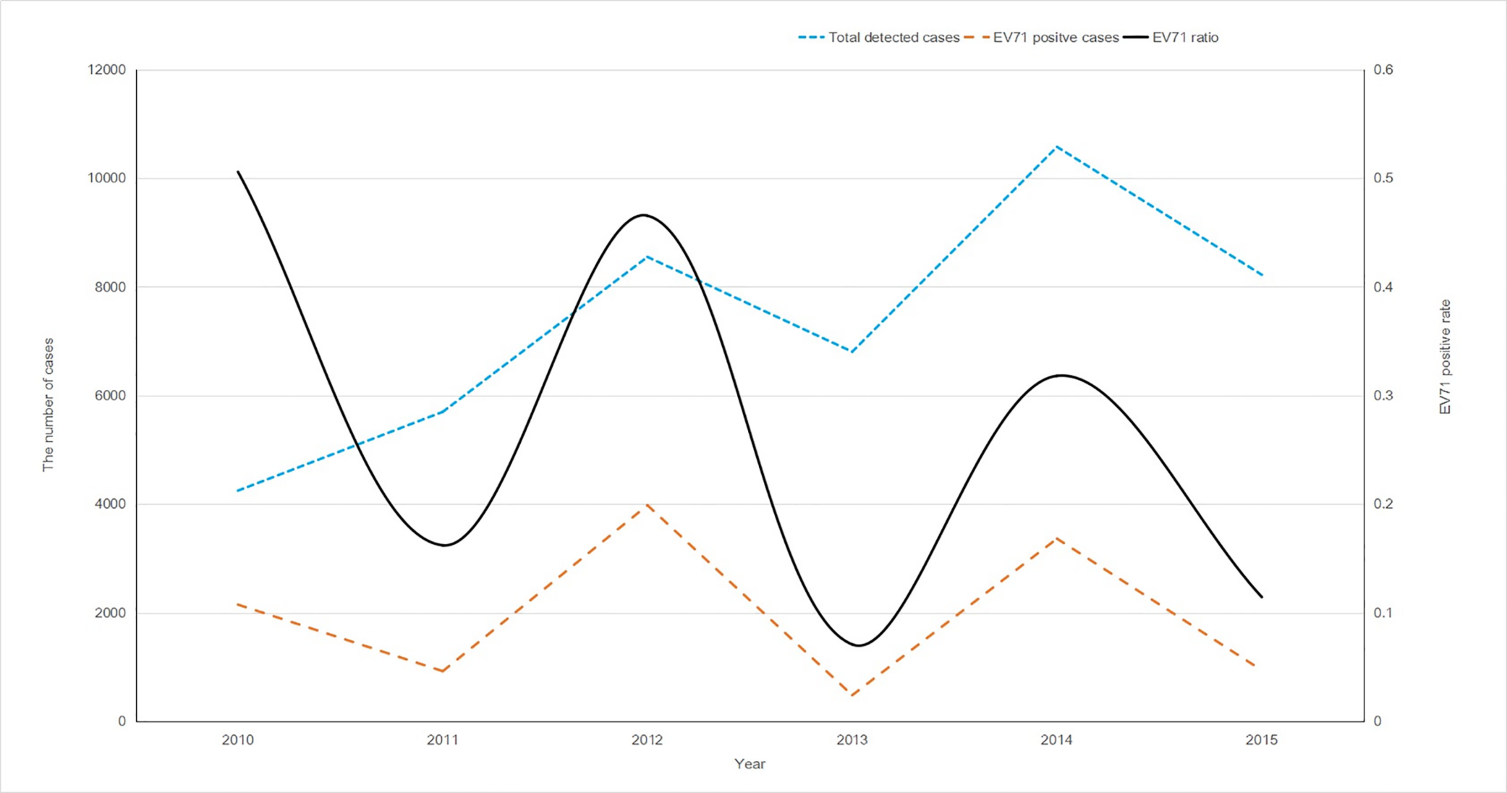
Yearly distribution (2010–2015) of laboratory-reported hand, foot, and mouth disease (HFMD) cases, *human enterovirus 71* (EV71)-positive cases, and EV71-positive rate, in Guangxi.

The total number of detected cases tended to increase from 2010 to 2015 in Guangxi (Fig 1). Meanwhile, the number of EV71-positive cases showed a wave-like distribution, with large oscillations occurring between years with large or small epidemics. Unexpectedly, the EV71-positive rate tended to decrease over the period of 2010–2015 (Fig 1). In general, there was an increase in the number of cases compared to the baseline from March to November.

**Fig 2:**
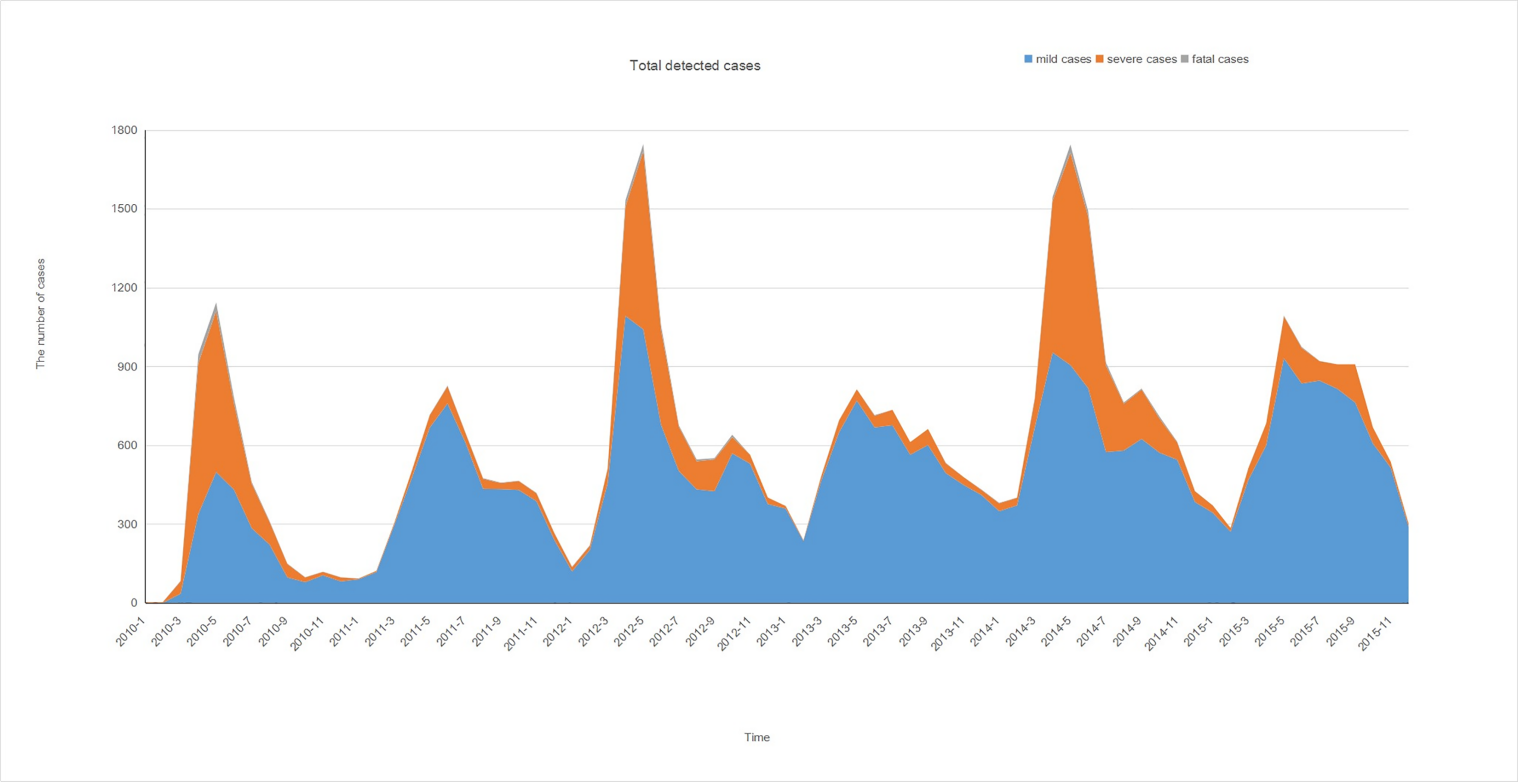
Monthly distribution of laboratory-reported hand, foot, and mouth disease (HFMD) cases in Guangxi from 2010 to 2015.

The prevalence of HFMD with semi-annual peaks in Guangxi was consistent with that of other southern Chinese provinces [16]; specifically, a major peak was observed from April to August, and another peak occurred from September to November. The summit of the major peak always occurred in May (Fig 2). In 2010, 2012, and 2014, there were sharper increases in the number of cases during April to October, than in 2011, 2013, and 2015. Both severe and fatal cases occurred mainly from April to June, whereas mild cases occurred throughout the entire year (Fig 2).

**Fig 3:**
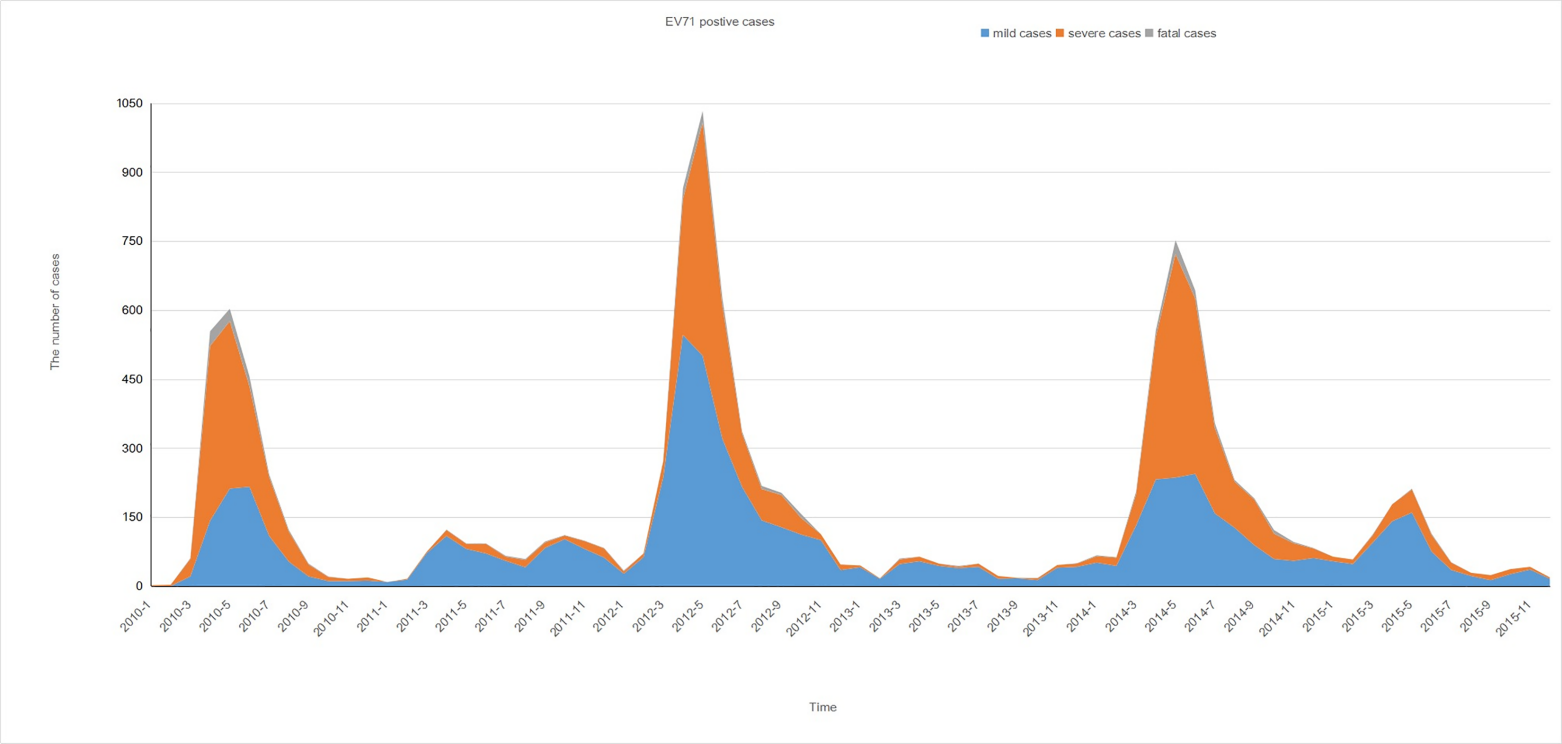
Distribution of *human enterovirus 71* (EV71)-positive hand, foot, and mouth disease (HFMD) cases in Guangxi from 2010 to 2015 by month.

**Fig 4:**
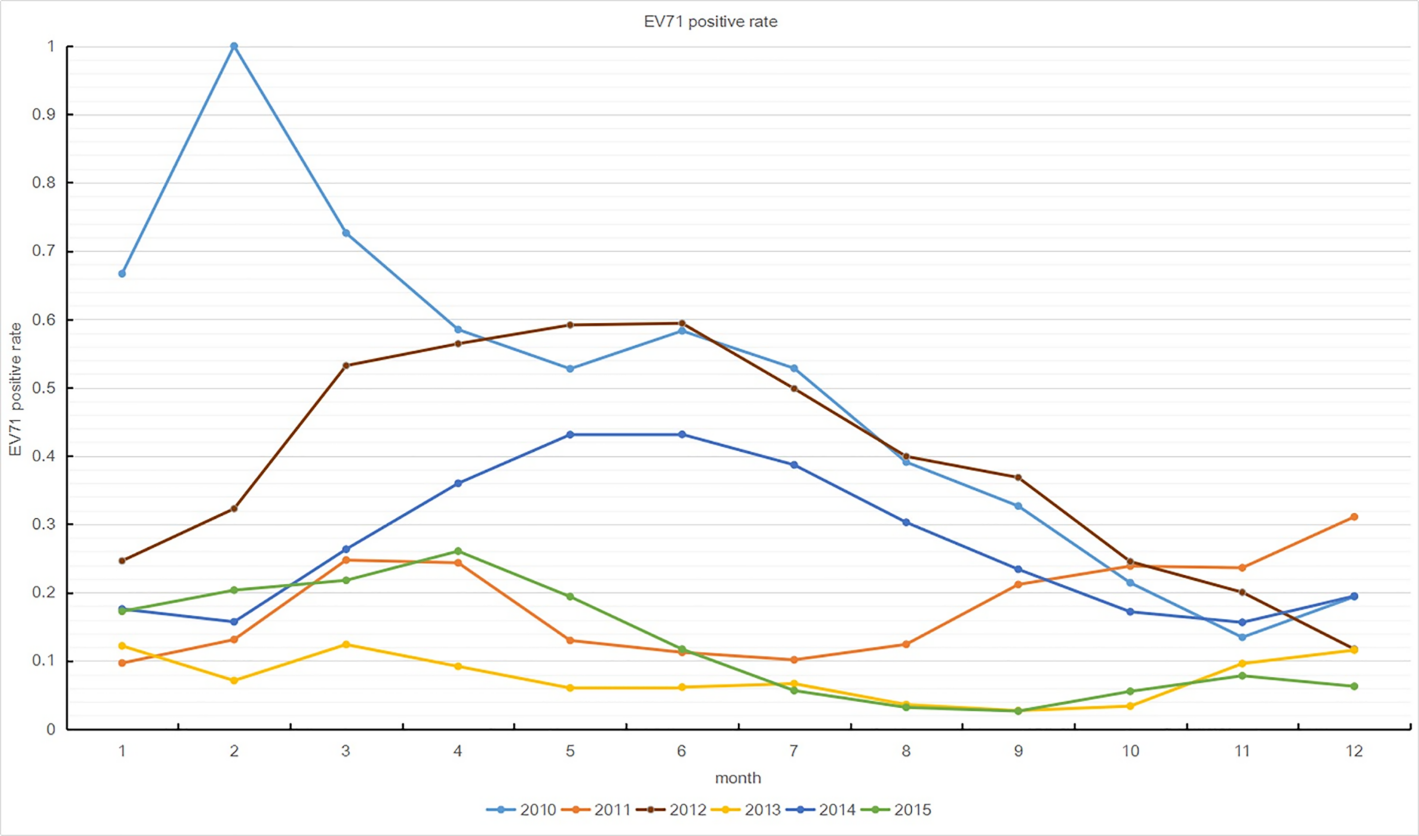
Distribution of *human enterovirus 71* (EV71)-positive rate in Guangxi from 2010 to 2015 by month.

There were drastic increases in the number of EV71-positive cases from April to July during 2010, 2012, and 2014, which were not observed in 2011, 2013, and 2015 (Fig 3). The mild cases were distributed year-round, whereas severe cases were found in the period of April to August. The EV71-positive rate was higher during the major peak than during the small peak, especially in large epidemic years (Fig 4). These data suggest that the EV71 was the dominant pathogen for HFMD cases that occurred from April to August, but not for HFMD cases that occurred from September to November.

### Pathogen detection

Of the 44,284 clinically diagnosed HFMD cases, a total of 33,498 (75.67%) were positive for enteroviruses, including 12,007 cases (27.11%) positive for EV71, 6,468 (14.61%) positive for CA16, and 15,023 positive (33.92%) for other enteroviruses. The positive rates of EV71, CA16, and other enteroviruses identified each year were significantly different (χ^2^ = 894.90, p < 0.001). Higher EV71-positive rates occurred in years with large epidemics (2010, 2012, and 2014), compared to those in years with small epidemics (2011, 2013, and 2015; p = 0.03725), and this was true for mild, severe, and fatal cases (Table 2).

**Table 2:** Distribution of *human enterovirus 71* (EV71)-positive cases in clinically diagnosed hand, foot, and mouth disease (HFMD) during 2010–2015.

Year	Mild cases			Severe cases			Fatal cases		
	EV71	Total	EV71 rate (%)	EV71	Total	EV71 rate (%)	EV71	Total	EV71 rate (%)
2010	834	2215	37.65	1246	1927	64.66	96	107	89.72
2011	820	5296	15.48	155	383	40.47	15	21	71.43
2012	2432	6384	38.10	1457	2063	70.63	97	103	94.17
2013	413	6378	6.48	63	417	15.11	6	11	54.55
2014	1481	7335	20.19	1595	3130	50.96	101	111	90.99
2015	732	7335	9.98	394	877	44.93	8	10	80.00

Compared to those for CA16 and other enteroviruses, the EV71-positive rates were significantly higher in severe cases (χ^2^ = 4591.13, p < 0.001) and fatal cases (χ^2^ = 706.89, p < 0.001) each year, whereas they were lower in mild cases (χ^2^ = 5282.46, p < 0.05). Thus, EV71 appears to be the dominant pathogen causing severe and fatal HFMD from 2010–2015.

### Geographical distribution of EV71-positive cases

**Fig 5:**
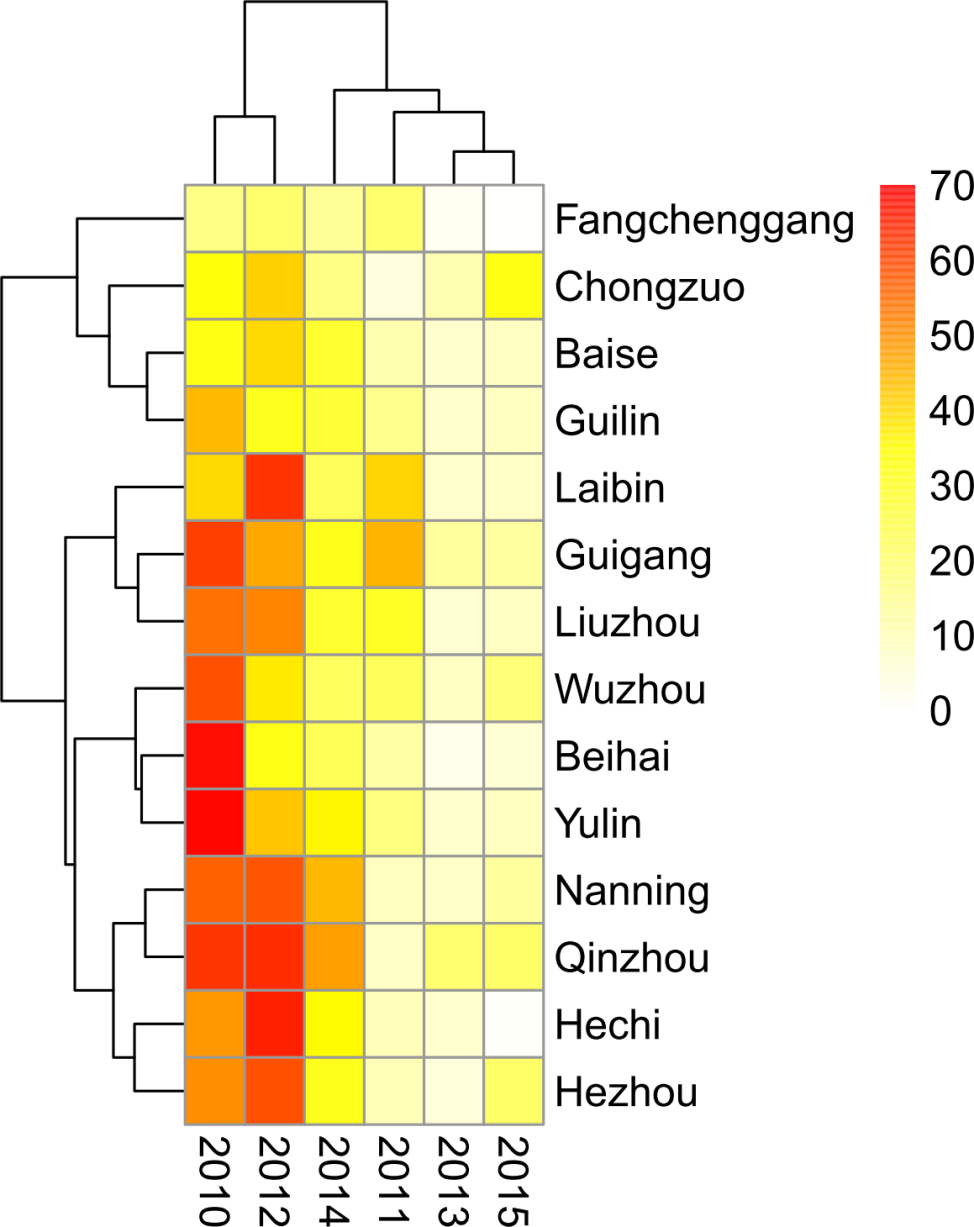
Heatmap of the yearly distribution of *human enterovirus 71* (EV71)-positive hand, foot, and mouth disease (HFMD) cases by city in Guangxi, 2010–2015.

From 2010 to 2015, there were differences in EV71-positive rates among the 14 cities in Guangxi each year. Four cities (Fangchenggang, Chongzuo, Baise, and Gulin) had lower EV71-positive rates, and four cities (Nanning, Qinzhou, Hechi, and Hezhou) had higher EV71-positive rates during the study years (Fig 5). In particular, a consistently low EV71-positive rate was found in Fangchenggang from 2010 to 2015.

**Fig 6:**
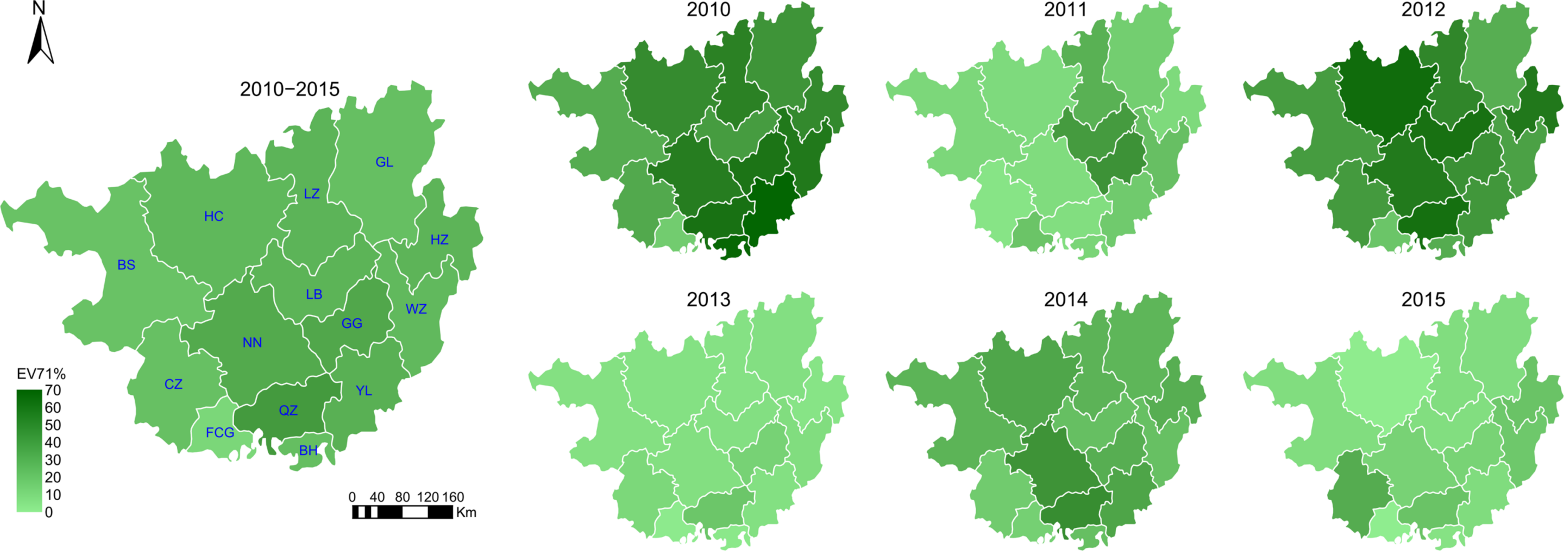
Annual *human enterovirus 71* (EV71)-positive rates for hand, foot, and mouth disease (HFMD) at the provincial municipality level in Guangxi, 2010–2015. BS: Baise; HC: Hechi; LZ: Liuzhou; GL: Guilin; CZ: Chongzuo; NN: Nanning; LB: Laibin; GG: Guigang; HZ: Hezhou; FCG: Fangchenggang; QZ: Qinzhou; BH: Beihai; YL: Yulin; WZ: Wuzhou.

The cities with the highest EV71-positive rates were not the same during each large epidemic. For example, in 2010, high EV71-positive rates were found in Beihai, Yulin, and Guigang, whereas in 2012, the highest rates were in Hechi and Laibin, with Qinzhou and Nanning having the highest rates in 2014 (Fig 6). Cities with the highest EV71 prevalence also alternated during each small epidemic. In 2011, high EV71-positive rates were noted in Laibin and Guigang, which are located in the middle of Guangxi. In 2013, EV71-positive rates for all 14 cites were relatively low (under 25%). In 2015, a high EV71-positive rate was noted in Chongzuo, a western city in Guangxi.

### Phylogenetic analysis of VP1 genes of EV71

**Fig 7:**
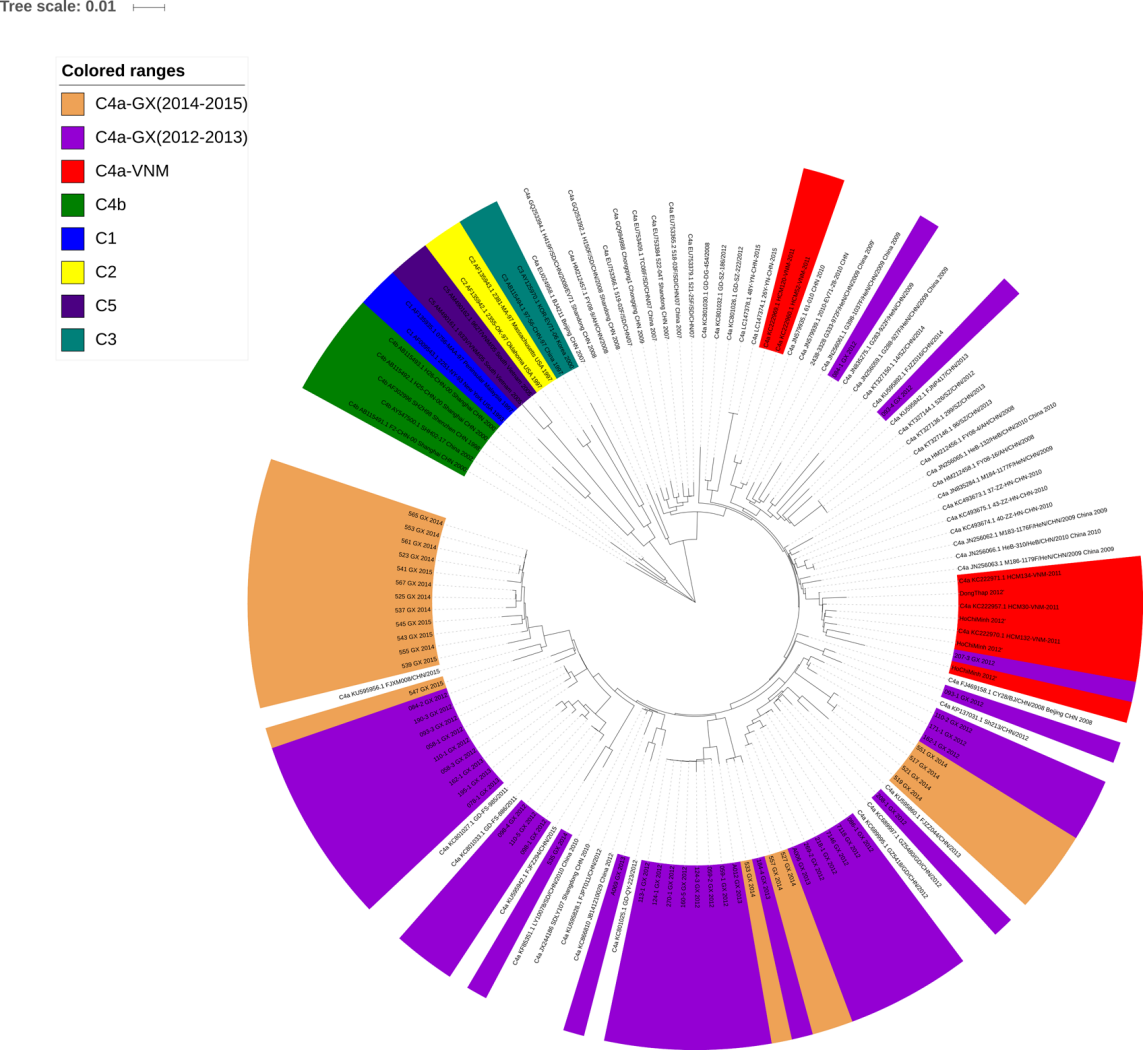
Phylogenetic analyses of *human enterovirus 71* (EV71) in Guangxi from 2010 to 2015.

A phylogenetic tree was constructed based on complete VP1 sequences (891 nucleotides) composed of the C subtypes (C1–C5) of EV71 strains from the present study and respective representative strains. Overall, VP1 genes of all EV71 strains identified in this study exhibited 95.2–100.0% nucleotide similarity, corresponding to 97.6–100% amino acid similarity. All of these strains clustered exclusively to genotype C, subtype 4a (C4a; Fig 7), which has been the dominant subtype circulating in mainland China in recent years.

Several strains isolated in 2012 and 2013 (in our study) displayed a close genetic relationship with other strains from Guangdong province, isolated in 2011 and 2012. Meanwhile, strains isolated from 2014 and 2015 were found to cluster with strains from Fujian province isolated in 2013 and 2015. Thus, the molecular characteristics of the EV71 strains tend to change gradually over time. Surprisingly, there was one strain, 207–3_GX_2012, that was phylogenetically closer to Vietnamese strains isolated in 2012 than to strains from mainland China.

## Discussion

Two-year HFMD epidemic cycles were observed in Guangxi, and were predominately caused by EV71; these were different from the three-year HFMD epidemic cycles that occurred in other areas in China such as Shijiazhuang city, which were primarily caused by EV71 and CA16 [17].

We found that the EV71-positive rate tended to decrease from 2010 to 2015. In years with large epidemics, such as 2014, the EV71-positive rate in clinically-diagnosed HFMD was found to decrease, compared to the rates in 2010 and 2012. There was also a similar trend for years with small epidemics; specifically, the EV71-positive rate was also found to be diminished in 2015, compared to that in 2011 and 2013. The fact that the EV71-positive rate tended to diminish was observed not only in Guangxi, but also in other provinces in China, according to the report from the Chinese Center for Disease Control and Prevention. Overall, the EV71-positive rate was reduced from 2010 to 2015, which mainly resulted from the decrease in mild cases associated with EV71 infection.

In December 2015, two inactivated EV71 vaccines, which were the first HFMD vaccines, were approved in mainland China for preventing severe HFMD [6, 7]. EV71 consists of one serotype with multiple sub-genotypes, and consequently expresses a variety of different antigens. Antibodies produced in response to an EV71 vaccine (EV71-H07, C4a strain) have been shown to cross-react with different EV71 genotypes including the B and C1–C4 sub-genotypes [18]; this should provide effective protection against HFMD caused by EV71. The epidemiological and genetic characterization of EV71 might now be affect by EV71 vaccination; therefore, there is still a need to monitor EV71 sequence variations to evaluate the genetic effects that occur after EV71 vaccination. The EV71 vaccine has been available in Guangxi since 2016. However, its relatively high cost, incurred by the recipients, has contributed to a low vaccine coverage level in Guangxi. EV71 was always the predominant pathogen for severe and fatal cases from 2010 to 2015. Thus, the EV71 vaccine is necessary across the province to abate disease burden. We suggest that EV71 vaccination should be prioritized in four cities with relatively high incidences of EV71 infection (Nanning, Qinzhou, Hechi, and Hezhou).

We predict that positive rates of non-EV71 and CA16 enterovirus such as CA6 and CA10 would be increased in mild cases in Guangxi from 2010 to 2015. For this, the results will be analyzed in a further study. It is now important to extend this pathogen detection to several other enteroviruses such as CA6 and CA10. Thus, these data suggest that HFMD pathogens other than EV71 are becoming more prevalent, and this fact should receive more attention.

In this study, we found that the EV71-positive rates for HFMD among 14 cities in Guangxi differed among cities each year. Regardless of large epidemic (2010, 2012, and 2014) or small epidemic (2011, 2013, and 2015) years, cities with the highest EV71-positive rates were not the same from year-to-year. One possible reason for this is that antibodies produced after EV71 infection might protect people from re-infection.

From 2010 to 2015, EV71 was one of the most important pathogens that caused HFMD in 14 Guangxi cities, with the exception of Fangchenggang city. Interestingly, we found that Fangchenggang always had the lowest EV71-positive rate from 2010 to 2015. Although it had a high incidence of HFMD, there was a low number of severe and fatal cases during this 6-year period. The EV71-positive rate was highest for fatal cases, which was followed by severe cases, and this was lowest in mild cases, which is consistent with previous results found in Henan and Hunan provinces [15–16]. The EV71-positive rate for mild cases in Fangchenggang was similar to that for mild cases in 13 cities. Thus, it appears that the few severe and fatal cases might be associated with a low EV71-positive rate in Fangchenggang; however, the reasons for this phenomenon require further exploration.

In China, the nationwide transmission of HFMD was reported to be associated with the relative humidity, birth rate, and population density. With respect to the latter, an interaction between the log of the population density and the Health System Performance has been observed using a multiple linear regression model [19]. Although it has a lower population density, Guangxi has relatively higher humidity and lower Health System Performance, which might facilitate the transmission of HFMD. However, we found four cities (Fangchenggang, Baise, Chongzuoin, and Gulin) that had a relatively low EV71 rate in HFMD from 2010 to 2015. The possible reasons for this might be related to the fact that that they had low levels of rainfall, a lower population density, and a lower temperature, compared to those in other cities in Guangxi.

In recent years, the EV71-C4 sub-genotype has been the major type of EV71 found in epidemics in mainland China. This subtype can be further divided into the C4b and C4a evolutionary branches, which correspond to two time periods [18]. The C4b evolutionary branch contains strains from Shenzhen and Shanghai, 1998–2004, and C4a has been identified in mainland China since 2003 [20, 21]. In this study, phylogenetic analysis of the VP1 gene showed that the EV71 strains sequenced in this study all belong to C4a, with most of them exhibiting higher identity to strains circulating in southern China (especially in Fujian and Guangdong provinces) than to strains in northern China. Moreover, the predominant circulating EV71 strains in Guangxi collected from 2012 to 2015 were phylogenetically distinct from the strains collected from China before 2008.

Interestingly, one strain, 207–3, which was isolated in 2012, was similar to Vietnamese strains isolated in 2011–2012. There is also a reference strain, HCM120/VNM/2011, isolated in Vietnam, which forms a closely-related cluster with mainland China strains. There is strong phylogenetic support suggesting that this lineage was likely derived from strains circulating in China in 2010, as has previously been described [22, 23]. Thus, these data suggest that a close interaction occurs between China and neighboring Vietnam.

Only a small number of EV71-positive HFMD specimens collected from Nanning city were used for cell culture and genetic analysis, but an EV71 stain was found to cluster with Vietnamese strains, suggesting that there is high risk of EV71 introduction from neighboring counties. Thus, surveillance of HFMD pathogens and the genetic analysis of the EV71 stain should be expanded in the three border cities (Chongzuo, Baise and Fangchenggang) of Guangxi, even though these were all associated with low EV71 infection rates.

## Supporting information

S1 Table GenBank accession numbers for the *human enterovirus 71* (EV71) sequences obtained in this study. (DOCX)
