# Supplementary material for: Epidemiological and genetic characteristics of EV71 in hand, foot, and mouth disease in Guangxi, southern China, from 2010 to 2015
Source: PLoS One. 2017 Dec 7;12(12):e0188640. doi: 10.1371/journal.pone.0188640 (PMC5720782; doi:10.1371/journal.pone.0188640)
Supplement: S1 Table — (DOCX) [file pone.0188640.s001.docx]

Supplementary Table 1. The GenBank accession numbers of EV71 obtained in this study.

| Subtype | GenBank accession No. | Name of strain | Geographic origin | Year of isolation |
| --- | --- | --- | --- | --- |
| C1 | AF009543.1 | 2251 | New York(USA) | 1993 |
| C1 | AF135935.1 | 0756 | Peninsular(Malaysia) | 1997 |
| C2 | AF135942.1 | 2355 | Oklahoma(USA) | 1997 |
| C2 | AF135943.1 | 2381 | Massachusetts(USA) | 1997 |
| C3 | AB115494.1 | 56 | CHN | 1997 |
| C3 | AY125970.1 | 06 | Korea | 2000 |
| C4a | FJ469158.1 | CY28 | Beijing(CHN) | 2008 |
| C4a | GQ253392.1 | H150F | Shandong(CHN) | 2008 |
| C4a | EU024958.1 | BJ4211 | Beijing(CHN) | 2007 |
| C4a | GQ253394.1 | H419F | Shandong(CHN) | 2008 |
| C4a | EU753384 | 522-04T | Shandong(CHN) | 2007 |
| C4a | GQ994988 | Chongqing1 | Chongqing(CHN) | 2009 |
| C4a | JX244186 | SDLY107 | Shangdong(CHN) | 2010 |
| C4a | KC866810 | JB141210029 | CHN | 2012 |
| C4a | KF85351.1 | LY10078 | Shandong(CHN) | 2010 |
| C4a | KU159520.1 | ND-181 | Vietnam:HoChiMinh | 2012 |
| C4a | KU159519.1 | ND-179 | Vietnam:HoChiMinh | 2012 |
| C4a | KU159515.1 | ND-174 | Vietnam:HoChiMinh | 2012 |
| C4a | KU159512.1 | ND-169 | Vietnam:DongThap | 2012 |
| C4a | EU753409.1 | TC08F | CHN | 2007 |
| C4a | JN256062.1 | M183-1176F | HeNan(CHN) | 2009 |
| C4a | JN256066.1 | HeB-310 | HeBei(CHN) | 2010 |
| C4a | JN256063.1 | M186-1179F | HeNan(CHN) | 2009 |
| C4a | JN256065.1 | HeB-132 | HeBei(CHN) | 2010 |
| C4a | JN256059.1 | G288-927F | HeNan(CHN) | 2009 |
| C4a | JN256060.1 | G333-972F | HeNan(CHN) | 2009 |
| C4a | JN256061.1 | G398-1037F | HeNan(CHN) | 2009 |
| C4a | EU753365.2 | 518-03F | Shandong(CHN) | 2007 |
| C4a | KC222970.1 | HCM132 | Vietnam | 2011 |
| C4a | KC222971.1 | HCM134 | Vietnam | 2011 |
| C4a | KC222957.1 | HCM30 | Vietnam | 2011 |
| C4a | KC222969.1 | HCM120 | Vietnam | 2011 |
| C4a | HM212458.1 | FY08-16 | AnHui(CHN) | 2008 |
| C4a | HM212457.1 | FY08-9 | AnHui(CHN) | 2008 |
| C4a | HM212456.1 | FY08-4 | AnHui(CHN) | 2008 |
| C4a | KU595828.1 | FJPT011 | CHN | 2012 |
| C4a | KP137031.1 | Sh213 | CHN | 2012 |
| C4a | KT327144.1 | 526 | Shenzhen(CHN) | 2012 |
| C4a | KU595860.1 | FJZZ044 | CHN | 2013 |
| C4a | KU595842.1 | FJNP417 | CHN | 2013 |
| C4a | KT327136.1 | 299 | Shenzhen(CHN) | 2013 |
| C4a | KU595892.1 | FJZZ016 | CHN | 2014 |
| C4a | KT327150.1 | 14 | Shenzhen(CHN) | 2014 |
| C4a | KT327146.1 | 96 | Shenzhen(CHN) | 2013 |
| C4a | KU595956.1 | FJXM008 | CHN | 2015 |
| C4a | KU595942.1 | FJFZ294 | CHN | 2015 |
| C4a | KC689997.1 | GZ5480 | GuangDong(CHN) | 2012 |
| C4a | KC689995.1 | GZ5418 | GuangDong(CHN) | 2012 |
| C4a | KC801033.1 | FS-886 | GuangDong(CHN) | 2011 |
| C4a | KC801032.1 | SZ-186 | GuangDong(CHN) | 2012 |
| C4a | KC801030.1 | DG-454 | GuangDong(CHN) | 2008 |
| C4a | KC801027.1 | FS-985 | GuangDong(CHN) | 2011 |
| C4a | KC801026.1 | SZ-222 | GuangDong(CHN) | 2012 |
| C4a | KC801025.1 | QY-223 | GuangDong(CHN) | 2012 |
| C4a | KC493675.1 | 43-ZZ | HeNan(CHN) | 2010 |
| C4a | KC493674.1 | 40-ZZ | HeNan(CHN) | 2010 |
| C4a | KC493673.1 | 37-ZZ | HeNan(CHN) | 2010 |
| C4a | LC147378.1 | 48Y | YunNan(CHN) | 2015 |
| C4a | LC147374.1 | 26Y | YunNan(CHN) | 2015 |
| C4a | JN835275.1 | G283-922F | HeNan(CHN) | 2009 |
| C4a | JN835284.1 | M184-1177F | HeNan(CHN) | 2009 |
| C4a | EU753379.1 | 521-25F | Shanghai(CHN) | 207 |
| C4a | EU753366.1 | 519-02F | Shanghai(CHN) | 2007 |
| C4a | JN579935.1 | 61 | CHN | 2010 |
| C4a | KC222960.1 | HCM52 | Vietnam | 2011 |
| C4a | JN579939.1 | 28 | CHN | 2010 |
| C4b | AB115491.1 | F2 | Shanghai(CHN) | 2000 |
| C4b | AY547500.1 | SHH02-17 | CHN | 2002 |
| C4b | AB115492.1 | H25 | Shanghai(CHN) | 2000 |
| C4b | AB115493.1 | H26 | Shanghai(CHN) | 2000 |
| C4b | AF302996 | SHZH98 | Shenzhen(CHN) | 1998 |
| C5 | AM490161.1 | 933V | South Vietnam | 2005 |
| C5 | AM490162.1 | 962T | South Vietnam | 2005 |
